# Fístula da Artéria Coronária: Associação entre Padrões de Trajetos, Características Clínicas e Cardiopatias Congênitas

**DOI:** 10.36660/abc.20190578

**Published:** 2021-07-15

**Authors:** Daniel L. Cobo, Fernando Batigalia, Ulisses A. Croti, Adilia M.P. Sciarra, Marcos H.D. Foss, Rafaela G.F. Cobo

**Affiliations:** 1 Faculdade de Medicina de São Jose do Rio PretoSão José do Rio PretoSPBrasilFaculdade de Medicina de São Jose do Rio Preto, São José do Rio Preto, SP - Brasil; 2 Hospital da Criança e Maternidade de São José do Rio PretoSão José do Rio PretoSPBrasilHospital da Criança e Maternidade de São José do Rio Preto, São José do Rio Preto, SP - Brasil

**Keywords:** Doença da Artéria Coronária, Fístula Artério-Arterial, Cardiopatias Congênitas, Anatomia, Dispnea, Cianose, Ecocardiografia/métodos, Imagem Tridimensional/métodos

## Abstract

**Fundamento:**

A fístula da artéria coronária (FAC) é uma conexão direta entre uma ou mais artérias coronárias e câmaras cardíacas ou um grande vaso; pode estar associada à cardiopatia congênita.

**Objetivo:**

Estabelecer os padrões de trajetos de FAC a partir de dados ecocardiográficos e correlacioná-los com aspectos clínicos e cardiopatias congênitas.

**Métodos:**

Um total de 7.183 prontuários médicos de crianças menores de 5 anos de idade com cardiopatia submetidas a ecodopplercardiograma colorido foram analisados utilizando o teste de correlação de Spearman para associar sinais, sintomas e cardiopatia à FAC, com nível de significância de 5%.

**Resultados:**

Vinte e seis crianças (0,0036%) apresentaram FAC, nos seguintes trajetos: da artéria coronária direita para o ventrículo direito (26,92%), da artéria coronária esquerda para o ventrículo direito (23,08%), do ramo interventricular anterior para o ventrículo direito (23,08%), da artéria coronária direita para o átrio direito (11,54%), da artéria coronária esquerda para o tronco pulmonar (7,69%) e do ramo interventricular anterior para o tronco pulmonar (7,69%). Em 57,69% dos pacientes, houve uma correlação positiva entre sintomas e a FAC (p = 0,445), relacionada à dispneia ou cianose (53,84%). Em 96,15%, a cardiopatia congênita estava associada à FAC; principalmente, a comunicação interventricular e a comunicação interatrial, em 34,62% dos casos, correlacionaram-se positivamente com a FAC (p = 0,295). O trajeto da FAC foi representado em três dimensões pelo software de modelagem, texturização e animação Cinema 4D R19.

**Conclusão:**

A FAC é uma entidade anatômica incomum que apresenta quadro clínico compatível com dispneia e cianose e está associada a cardiopatias congênitas, principalmente com a CIV ou a CIA. De acordo com as análises ecocardiográficas, as fístulas na ACD, na ACE ou no RIVA representam aproximadamente um terço dos pacientes, com trajeto prioritário para as câmaras cardíacas direitas.

## Introdução

A fístula da artéria coronária (FAC) é uma conexão direta entre uma ou mais artérias coronárias a câmaras cardíacas ou a um grande vaso. É uma das anomalias da artéria coronária mais comuns, embora seja considerada rara na população em geral.^1,2^ Está presente em 0,002% da população e representa 0,4% de todas as malformações cardíacas.^3,4^

O ecocardiograma Doppler tem sido indicado para avaliação de cardiopatias congênitas devido a sua versatilidade diagnóstica, disponibilidade, custo-efetividade e quantidade de informações morfofuncionais que fornece para o coração,^5^

Devido à raridade da FAC e à potencial contribuição da sua caracterização topográfica, o presente estudo visou determinar os padrões de trajetos da FAC a partir de dados ecocardiográficos e correlacioná-los com aspectos clínicos e cardiopatias congênitas.^6,7^

## Método

Após a aprovação ética, foram considerados 7.183 prontuários eletrônicos de pacientes pediátricos com ou sem cardiopatia congênita do Serviço de Cardiologia e Cirurgia Cardiovascular Pediátrica do Hospital de Base e do Hospital da Criança e Maternidade de São José do Rio Preto, São Paulo, Brasil. Foram realizados exames ecocardiográficos bidimensionais em cores (Philips Healthcare® modelos HD 11 e HD 15) de acordo com as diretrizes da Sociedade Americana de Ecocardiografia.^8^ Crianças com outros defeitos cardíacos congênitos que não FAC e crianças com mais de cinco anos de idade devido a cardiopatia congênita sob cuidados durante os primeiros meses até os primeiros anos de vida foram excluídos do estudo. Foi definida por conveniência o tamanho da amostra do presente estudo.

### Análise estatística

Foram obtidas as análises estatísticas utilizando o software SPSS Statistics versão 23.0^9^ e Excel (versão 2.016). As variáveis categóricas consideradas no presente estudo compreenderam os seguintes sinais e sintomas: assintomático, baixa saturação de O_2_, cianose (das extremidades, labial ou generalizada), hipertermia e dispneia; as seguintes cardiopatias associadas: atresia pulmonar, coarctação da aorta, comunicação interatrial (CIA) ou comunicação interventricular (CIV), defeito do septo atrioventricular, estenose da válvula pulmonar, ducto arterial persistente, tetralogia de Fallot e sem cardiopatia associada; bem como a topografia da FAC a partir da descrição de relatórios ecocardiográficos para reconstrução tridimensional por pelo software de modelagem, texturização e animação Cinema 4D R19.^10^ A normalidade dos dados foi verificada por meio do teste de Shapiro-Wilk, que apresentou dados não paramétricos. Posteriormente, foi aplicado o teste de correlação de Spearman para associar as cardiopatias congênitas e os sinais e sintomas com a FAC, estabelecendo valor de < 0,05.^9^

## Resultados

No presente estudo, foi apresentada uma análise descritiva (Tabela 1) e, para as variáveis categóricas, foi analisado o cruzamento inferencial (Tabelas 2 e 3). Foi utilizado o teste de correlação de Spearman e foi apresentado um único valor de p para cada cruzamento. Dos 7.183 prontuários considerados, foi detectada a FAC em 26 casos (0,0036%). A Tabela 1 mostra as variáveis categóricas de trajetos de FAC detectados por ecocardiograma, demonstrando que as fístulas na artéria coronária direita (ACD), na artéria coronária esquerda (ACE) ou no ramo interventricular anterior (RIVA) representaram aproximadamente um terço dos pacientes, com trajeto prioritário para as câmaras cardíacas direitas.

**Tabela 1:** – Variáveis categóricas de trajetos de fístula da artéria coronária detectados por ecocardiograma

Tipo de fistula	N	%
ACD para VD	7	26,92
ACD para AD	3	11,54
ACE para TP	2	7,69
ACE para VD	6	23,08
RIVA para TP	2	7,69
RIVA para VD	6	23,08
**Total**	**26**	**100**

*ACD: artéria coronária direita, ACE: artéria coronária esquerda; AD: átrio direito; N: valor absoluto; RIVA: ramo interventricular anterior; TP: tronco pulmonar; VD: ventrículo direito.*

A Tabela 2 correlacionou as variáveis categóricas, os sinais e sintomas (assintomáticos, baixa saturação de O_2_, cianose das extremidades, labial ou generalizada, hipertermia e dispneia) com os tipos da FAC (ACD, ventrículo direito, átrio direito, ACE, tronco pulmonar e RIVA) e mostra que 26,92% dos pacientes com FAC eram assintomáticos e 57,69% eram sintomáticos, expressos por dispneia (26,92%), cianose (26,92%) e hipertermia (3,86%). A baixa saturação de oxigênio foi detectada pelo oxímetro de pulso em 15,38% dos pacientes. A partir desses dados, foi realizado o teste de correlação de Spearman, que observou valor de p = 0,445, demonstrando não haver evidência estatística de dependência entre as variáveis analisadas.

**Tabela 2:** – Correlação das variáveis categóricas de sinais e sintomas com os tipos de fístula da artéria coronária

Sinais e sintomas	Tipos de fístula da artéria coronária												TOTAL	
	ACD/VD		ACD/AD		ACE/TP		ACE/VD		RIVA/TP		RIVA/VD			
	N	%	N	%	N	%	N	%	N	%	N	%	N	%
Assintomático	0	0	2	66,67	1	50	2	33,33	0	0	2	33,33	7	26,92
Baixa saturação de O_2 _	2	28,57	0	0	0	0	2	33,33	0	0	0	0	4	15,38
Cianose das extremidades, labial ou generalizada	3	42,86	0	0	0	0	0	0	1	50	3	50	7	26,92
Hipertermia	0	0	0	0	0	0	0	0	0	0	1	16,67	1	3,86
Dispneia	2	28,57	1	33,33	1	50	2	33,33	1	50	0	0	7	26,92
**Total**	**7**	**100**	**3**	**100**	**2**	**100**	**6**	**100**	**2**	**100**	**6**	**100**	**26**	**100**
**Valor (p)**													**0,445**	

*ACD: artéria coronária direita; ACE: artéria coronária esquerda; AD: átrio direito; N: valor absoluto; RIVA: ramo interventricular anterior; TP: tronco pulmonar; VD: ventrículo direito.*

A Tabela 3 mostra que a FAC esteve associada à cardiopatia congênita em 96,15% dos casos, principalmente com a CIV ou a CIA em 34,62% dos casos, a tetralogia de Fallot (23,08%) e a coarctação de aorta (11,53%). As variáveis categóricas de cardiopatia associada (atresia pulmonar, coarctação da aorta, CIA ou CIV, defeito do septo atrioventricular, estenose da válvula pulmonar, ducto arterioso persistente, tetralogia de Fallot e sem cardiopatia associada) foram correlacionadas com os tipos de FAC (ACD, ventrículo direito, átrio direito, ACE, tronco pulmonar e RIVA) e, a partir desses dados, foi realizado o teste de correlação de Spearman, que observou valor de p = 0,295, demonstrando não haver evidência estatística de dependência entre as variáveis analisadas.

**Tabela 3:** – Correlação das variáveis categóricas de cardiopatia associada com os tipos de fístula da artéria coronária

Doença cardíaca associada	Tipos de fístula da artéria coronária												TOTAL	
	ACD/VD		ACD/AD		ACE/TP		ACE/VD		RIVA/TP		RIVA/VD			
	N	%	N	%	N	%	N	%	N	%	N	%	N	%
Atresia pulmonar	1	14,29	0	0	0	0	0	0	0	0	0	0	1	3,85
Coarctação da aorta	0	0	0	0	1	50	0	0	1	50	1	16,67	3	11,53
CIA ou CIV	2	28,57	2	66,67	0	0	2	33,33	0	0	3	50	9	34,62
Defeito do septo atrioventricular	0	0	0	0	0	0	0	0	0	0	1	16,67	1	3,85
Estenose da válvula pulmonar	1	14,29	0	0	0	0	0	0	0	0	1	16,67	2	7,69
Sem cardiopatia associada	0	0	1	33,33	1	50	1	16,67	0	0	0	0	3	11,53
Ducto arterioso persistente	0	0	0	0	0	0	0	0	1	50	0	0	1	3,85
Tetralogia de Fallot	3	42,86	0	0	0	0	3	50	0	0	0	0	6	23,08
**Total**	**7**	**100**	**3**	**100**	**2**	**100**	**6**	**100**	**2**	**100**	**6**	**100**	**26**	**100**
**Valor (p)**													**0,295**	

*ACD: artéria coronária direita; ACE: artéria coronária esquerda; AD: átrio direito; CIA: comunicação interatrial; CIV: comunicação interventricular; N: valor absoluto; RIVA: ramo interventricular anterior; TP: tronco pulmonar; VD: ventrículo direito.*

**Figura 1:**
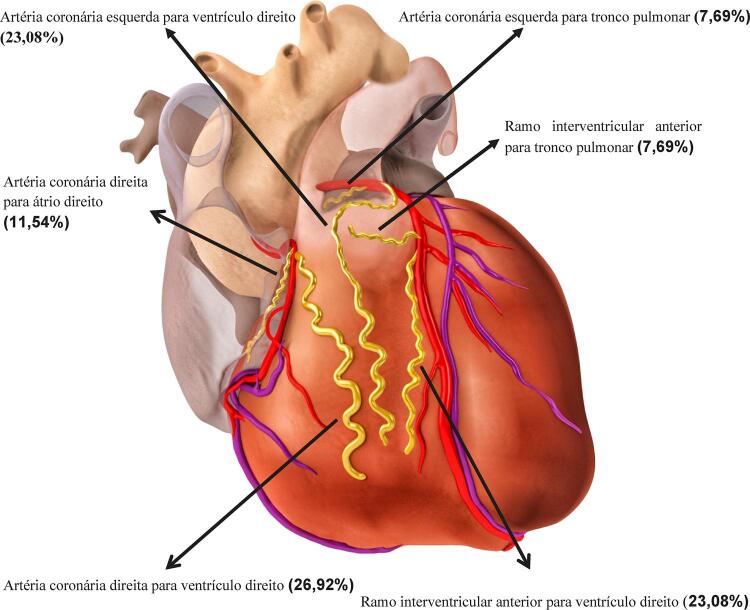
– Representação topografia de trajetos de fístula da artéria coronária e seus percentuais respectivos.

A Figura 1 mostra os padrões de trajeto encontrados para a FAC e os seus percentuais respectivos.^10^

## Discussão

No presente estudo, a prevalência de FAC foi observada em 0,0036% dos casos, embora a frequência real de anomalias da artéria coronária na população geral possa ser desconhecida ou extremamente rara.^11^ A prevalência estimada varia de 0,002%^12^ a 0,1% ou de 1% a 2%,^13,14^ e representa 14% de todas as anomalias da artéria coronária.^15^

Quase um terço dos pacientes eram assintomáticos; os sinais e sintomas foram representados por dispneia, cianose e hipertermia em 57,69% deles (Tabela 2). Embora a ausência de sintomas possa ser ainda mais prevalente, a angina devido ao fenômeno de “roubo coronário” (débito cardíaco diminuído), a endocardite, o infarto agudo do miocárdio ou a insuficiência cardíaca podem estar presentes.^14,16^ As crianças com FAC são frequentemente assintomáticas; portanto, estima-se que aproximadamente 80% dos pacientes menores de 20 anos de idade sejam assintomáticos.^17^

No presente estudo, a maioria das crianças (96,15%) com FAC apresentava outra cardiopatia congênita associada, principalmente CIV ou CIA, em um terço dos pacientes (Tabela 3). No entanto, a FAC como uma manifestação isolada pode estar presente em 55% a 80% dos casos e foi associada à cardiopatia congênita (tetralogia de Fallot, ducto arterioso persistente, CIV ou CIA) em 20% a 45%^17^ e doença arterial coronariana em até 35% das ocorrências.^18^ Além disso, a FAC frequentemente tem etiologia congênita, mas pode ser secundária a lesão, infecção, causas iatrogênicas ou doença de Kawasaki.^12^

De acordo com as análises ecocardiográficas, os resultados destacaram que as fístulas na ACD, na ACE ou no RIVA representam aproximadamente um terço dos pacientes, com trajeto prioritário para as câmaras cardíacas direitas (Tabela 1). Manoly et al., relataram que a FAC é mais prevalente na ACD (52% dos casos avaliados), com drenagem para o lado direito do coração em mais de 90% dos casos, o que foi corroborado por outros autores.^16^

A ecocardiografia bidimensional colorida tem sido recomendada para avaliar a FAC, embora a angiografia,^1,16^ o ecocardiograma transtorácico e a angiotomografia computadorizada apresentem resultados eficazes.^2,19^ Uma possível limitação do presente estudo refere-se à maior prevalência da FAC na ACD do que no átrio direito, possivelmente decorrente da forma iatrogênica após procedimento cirúrgico para correção da tetralogia de Fallot.^20,21^

Devido à baixa prevalência da FAC, está disponível um número reduzido de publicações científicas sobre o assunto. A contribuição do presente estudo aponta para o tamanho relativamente grande da amostra (26 registros) para uma anomalia cardíaca rara.^1,4^ Enquanto as análises ecocardiográficas nos prontuários eletrônicos apresentam representação bidimensional, a análise tridimensional realizada neste estudo pode ser mais eficaz.^22^ Os esforços para melhorar a imagem para FAC, de preferência tridimensional, podem melhorar o tratamento clínico, o planejamento cirúrgico e a intervenção intraoperatória.^23^

## Conclusões

A FAC é uma entidade anatômica incomum que apresenta quadro clínico compatível com dispneia e cianose e está associada a cardiopatias congênitas, principalmente com a CIV ou a CIA. De acordo com as análises ecocardiográficas, as fístulas na ACD, na ACE ou no RIVA estão presentes em aproximadamente um terço dos pacientes, com trajeto prioritário para as câmaras cardíacas direitas.
