# Supplementary material for: Fístula da Artéria Coronária: Associação entre Padrões de Trajetos, Características Clínicas e Cardiopatias Congênitas
Source: Arq Bras Cardiol. 2021 Jul 15;117(1):84–8. [Article in Portuguese] doi: 10.36660/abc.20190578 (PMC8294727; doi:10.36660/abc.20190578)
Supplement: Translation

# Translation: Coronary Artery Fistula: Association between Pathway Patterns, Clinical Features and Congenital Heart Disease

**Authors:** Daniel L. Cobo, Fernando Batigalia, Ulisses A. Croti, Adilia M.P. Sciarra, Marcos H.D. Foss, Rafaela G.F. Cobo

**Affiliations:** 1 BrasilFaculdade de Medicina de São Jose do Rio Preto, São José do Rio Preto, SP - Brasil; 2 BrasilHospital da Criança e Maternidade de São José do Rio Preto, São José do Rio Preto, SP - Brasil

**Keywords:** Coronary Artery Disease, Arterio-Arterial Fistula, Congenital Heart Diseases, Anatomy, Dyspnea, Cyanosis, Echocardiography/methods, Imaging Three-Dimensional/methods

## Abstract

**Background:**

Coronary artery fistula (CAF) is a direct connection of one or more coronary arteries to cardiac chambers or a large vessel; it may be associated with congenital heart disease.

**Objective:**

To establish CAF pathway patterns from echocardiographic data and to correlate them with clinical aspects and congenital heart disease.

**Methods:**

A total of 7.183 medical records of children under the age of five years with cardiac disease submitted to color Doppler echocardiography and Spearman’s Correlation test were used to associate signs and symptoms and cardiopathy to CAF with a significance level of 5%.

**Results:**

Twenty six children (0.0036%) presented CAF: from the right coronary artery (RCA) to the right ventricle (RV) 26.92%, from the left coronary artery (LCA) to the RV 23.08%, from the anterior interventricular branch (AIVB) to RV 23.08%, RCA to right atrium (RA) 11.54%, LCA for pulmonary trunk (PT) 7.69% or AIVB for PT 7.69%. In 57.69% of the patients, there was a positively correlated symptomatology to CAF with p=0.445 related to dyspnea or cyanosis (53.84%); in 96.15%, congenital heart disease associated with CAF, mainly interventricular communication (IVC) or interatrial communication (IAC) in 34.62% positively correlated to CAF with p=0.295. CAF pathway was represented in three dimensions by software modeling, texturing and animation Cinema 4D R19.

**Conclusion:**

CAF is an uncommon anatomical entity that presents a clinical picture compatible with dyspnea and cyanosis, and this is associated with congenital heart disease, mainly with IVC or IAC. According to echocardiographic analyzes, fistulas in RCA, LCA, or AIVB represent about one-third of the patients, with a priority pathway for right heart chambers.

## Introduction

Coronary artery fistula (CAF) is a direct connection of one or more coronary arteries to cardiac chambers or a large vessel. It is one of the most common coronary artery (CA) anomalies, although, it is considered rare in general population.^1,2^ It is present in 0.002% of the population, and it represents 0.4% of all cardiac malformations.^3,4^

Doppler echocardiogram has been indicated for the evaluation of congenital heart defects due to its diagnostic versatility, availability, cost-effectiveness and the amount of morphofunctional information of the heart.^5^

Due to the rarity of CAF and the potential contribution of its topographic characterization, the present study aims at determining CAF pathway patterns from echocardiographic data as well as to correlate them with clinical aspects and congenital heart disease.^6,7^

## Method

After ethical approval, 7,183 electronic medical records of pediatric patients with or without congenital heart disease from the Cardiology and Pediatric Cardiovascular Surgery Service of Hospital de Base (HB) and the Children Hospital and Maternity Hospital (CHMH) of São José do Rio Preto – SP – Brazil were considered. Two-dimensional color echocardiographic examinations (Philips Healthcare® HD 11 and HD 15 model) were performed according to the guidelines of the American Society of Echocardiography.^8^ Children with congenital heart defects without CAF, and children over five years of age due to congenital heart defect under care during initial months up to the first years of life were excluded from the study. The sample size used in the study was defined by convenience.

### Statistical Analysis

The statistical analyzes were obtained using the SPSS Statistics software version 23.0^9^ and the Excel tool (version 2.016). Variables considered in the study are categorical and comprised signs and symptoms such as: asymptomatic, low O_2_ saturation, cyanosis of the extremities, labial or generalized, hyperthermia and dyspnea; cardiopathy associated with: Pulmonary Atresia, Coarctation of the Aorta, IAC or IVC, Atrioventricular Septal Defect, Pulmonary Valve Stenosis, Persistence Ductus Arteriosus, Tetralogy of Fallot or no associated heart disease, and CAF topography from the description of echocardiographic reports for three-dimensional reconstruction by modeling, texturing and animation software Cinema 4D R19.^10^ The normality of the data was verified using the Shapiro-Wilk test, which showed nonparametric data. Afterwards, the Spearman’s Correlation test was applied to associate congenital heart disease and signs and symptoms to the CAF, which observed p value <0.05.^9^

## Results

In the present study, a descriptive analysis was presented (Table 1), and for categorical variables, inferential crossover was analyzed (Tables 2 and 3). Spearman’s correlation test was used, and for each crossing a single p value was presented. From the 7,183 medical records considered, CAF was detected in 26 cases (0.0036%). Table 1 shows categorical variables of pathways for CAF detected according to the echocardiogram, and shows that fistulas in the right coronary artery (RCA), left coronary artery (LCA) or in the anterior interventricular branch (AIVB) represent about one third of the patients, with priority pathway for the right heart chambers.

**Table 1:** – Categorical variables of coronary artery fistula pathways detected by echocardiogram

Type of fistula	N	%
RCA for RV	7	26.92
RCA for RA	3	11.54
LCA for PT	2	7.69
LCA for RV	6	23.08
AIVB for PT	2	7.69
AIVB for RV	6	23.08
**Total**	**26**	**100**

*AIVB: anterior interventricular branch, LCA: left coronary artery, N: absolute value, PT: pulmonary trunk, RA: right atrium, RCA: right coronary artery, RV: right ventricle.*

Table 2 correlated categorical variables, signs and symptoms (asymptomatic, low O_2_ saturation, cyanosis of the extremities, labial or generalized, hyperthermia and dyspnea) with types of CAF (RCA, RV, RA, LCA, PT and AIVB) and shows that in patients with CAF asymptomatology is present in 26.92% of the patients and symptomatology in 57.69%, expressed by dyspnea (26.92%), cyanosis (26.92%) and hyperthermia (3.86%). Low oxygen saturation detected by pulse oximeter; in 15.38% of the patients. From these data, the Spearman Correlation Test was performed, which observed p value = 0.445 and it is understood that there is no statistical evidence of dependence between the variables analyzed.

**Table 2:** – Correlation of the categorical variables of signs and symptoms with the types of coronary artery fistula

Signs and symptoms	Types of coronary artery fistula												TOTAL	
	RCA/RV		RCA/RA		LCA/PT		LCA/RV		AIVB/PT		AIVB/RV			
	N	%	N	%	N	%	N	%	N	%	N	%	N	%
Asymptomatic	0	0	2	66.67	1	50	2	33.33	0	0	2	33.33	7	26.92
Low O_2 _saturation	2	28.57	0	0	0	0	2	33.33	0	0	0	0	4	15.38
Extremities, labial, or generalized cyanosis	3	42.86	0	0	0	0	0	0	1	50	3	50	7	26.92
Hyperthermia	0	0	0	0	0	0	0	0	0	0	1	16.67	1	3.86
Dyspnea	2	28.57	1	33.33	1	50	2	33.33	1	50	0	0	7	26.92
Total	7	100	3	100	2	100	6	100	2	100	6	100	26	100
Value (p)													0.445	

*AIVB: anterior interventricular branch, LCA: left coronary artery, N: absolute value, PT: pulmonary trunk, RA: right atrium, RCA: right coronary artery, RV: right ventricle.*

Table 3 shows that CAF and associated congenital heart disease in 96.15% of the cases; mainly with interventricular communication (IVC) or interatrial communication (IAC) in 34.62% of the cases, tetralogy of Fallot (23.08%) and coarctation of aorta (11.53%). The categorical variables Associated Heart Disease (Pulmonary Atresia, Coarctation of the Aorta, IAC or IVC, Atrioventricular Septal Defect, Pulmonary Valve Stenosis, no associated heart disease, Persistence Ductus Arteriosus, Tetralogy of Fallot) were correlated with the types of CAF (RCA, RV, RA, LCA, PT and AIVB) and from these data, the Spearman Correlation Test was performed, which observed p value = 0.295 and it is understood that there is no statistical evidence of dependence between the variables analyzed.

**Table 3:** – Correlation of categorical variables of associated heart disease with types of CAF

Associated heart disease	Types of coronary artery fistula												TOTAL	
	RCA/RV		RCA/RA		LCA/PT		LCA/RV		AIVB/PT		AIVB/RV			
	N	%	N	%	N	%	N	%	N	%	N	%	N	%
Pulmonary atresia	1	14.29	0	0	0	0	0	0	0	0	0	0	1	3.85
Coarctation of the aorta	0	0	0	0	1	50	0	0	1	50	1	16.67	3	11.53
IAC or IVC	2	28.57	2	66.67	0	0	2	33.33	0	0	3	50	9	34.62
Atrioventricular septal defect	0	0	0	0	0	0	0	0	0	0	1	16.67	1	3.85
Pulmonary valve stenosis	1	14.29	0	0	0	0	0	0	0	0	1	16.67	2	7.69
No associated heart disease	0	0	1	33.33	1	50	1	16.67	0	0	0	0	3	11.53
Persistent ductus arteriosus	0	0	0	0	0	0	0	0	1	50	0	0	1	3.85
Tetralogy of Fallot	3	42.86	0	0	0	0	3	50	0	0	0	0	6	23.08
**Total**	**7**	**100**	**3**	**100**	**2**	**100**	**6**	**100**	**2**	**100**	**6**	**100**	**26**	**100**
**Value (p)**													**0.295**	

*AIVB: anterior interventricular branch; IAC: interatrial communication; IVC: interventricular communication; LCA: left coronary artery; N: absolute value; PT: pulmonary trunk; RA: right atrium; RCA: right coronary artery; RV: right ventricle.*

The Figure 1 shows the pathway patterns found for CAF and their respective percentages.^10^

**Figure 1:** – Topographic representation of coronary artery fistula pathways and their respective percentages.

## Discussion

In this study, CAF prevalence was observed in 0.0036% of the cases. Although the real frequency of coronary artery anomalies in the general population could be unknown or extremely rare.^11^ Estimated prevalence ranges from 0.002%^12^ to 0.1% to 1 or from 1% to 2%^13,14^ and represents 14% of all coronary artery anomalies.^15^

Asymptomatology was present in almost a third of the patients; signs and symptoms were represented by dyspnea, cyanosis and hyperthermia in 57.69% of the patients (Table 2). Although the lack of symptoms could be even more prevalent, angina due to the phenomenon of “coronary steal” (decreased cardiac output), endocarditis, acute myocardial infarction or heart failure could be present.^14,16^ Children with CAF are often asymptomatic, therefore, it is estimated that about 80% of patients under 20 years of age are asymptomatic.^17^

Most of the children (96.15%) of the study had another congenital heart disease associated with CAF, mainly IVC or IAC in one third of the patients (Table 3). However, CAF as an isolated manifestation could be present in 55% to 80% of the cases, and it was associated with congenital heart disease (Tetralogy of Fallot, persistence of the ductus arterious, IVC or IAC) in 20% to 45%,^17^ or coronary artery disease in up to 35% of the occurrences.^18^ In addition, CAF has frequently congenital etiology, but could be secondary to injury, infection, iatrogenic or Kawasaki disease.^12^

According to echocardiographic analyzes, the results have stood out that fistulas in RCA, LCA or AIVB represent about one third of the patients, with priority pathway for right cardiac chambers (Table 1). Manoly et al. reported that CAF is more prevalent in RCA (52% of the cases evaluated), with drainage to the right side of the heart in more than 90% of the cases, which has been corroborated by other authors.^16^

Two-dimensional color Echocardiography has been recommended to evaluate CAF, although angiography,^1,16^transthoracic echocardiogram or computerized angiotomography present effective results.^2,19^ A possible limitation of this study refers to a further higher CAF prevalence of RCA to RA, resulting from iatrogenic form after a surgical procedure to correct Tetralogy of Fallot.^20,21^

Because of the low prevalence of CAF, reduced amount of related scientific publications on the subject are available. The contribution of the present study points out to the relatively high sample size (26 records) for a rare cardiac anomaly.^1,4^ Even though echocardiographic analyzes on electronic records present two-dimensional representation; the three-dimensional analysis performed in this study could be more effective.^22^ Efforts to improve CAF imaging, preferably in three dimensions, can improve clinical treatment, surgical planning or intraoperative intervention.^23^

## Conclusions

CAF is an unusual anatomical entity that presents a clinical picture compatible with dyspnea and cyanosis, and it is associated with congenital heart disease, mainly with IVC or IAC. According to echocardiographic analyzes, fistulas in RCA, LCA, or AIVB are present in about one-third of the patients, with a priority pathway to right heart chambers.
